# N-Acetyl-L-cysteine Protects Human Retinal Pigment Epithelial Cells from Oxidative Damage: Implications for Age-Related Macular Degeneration

**DOI:** 10.1155/2019/5174957

**Published:** 2019-08-14

**Authors:** Marcia R. Terluk, Mara C. Ebeling, Cody R. Fisher, Rebecca J. Kapphahn, Ching Yuan, Reena V. Kartha, Sandra R. Montezuma, Deborah A. Ferrington

**Affiliations:** ^1^Department of Ophthalmology and Visual Neurosciences, University of Minnesota, Minneapolis, Minnesota, USA; ^2^Center for Orphan Drug Research, Department of Experimental and Clinical Pharmacology, University of Minnesota, Minneapolis, Minnesota, USA; ^3^Graduate Program in Biochemistry, Molecular Biology and Biophysics, University of Minnesota, Minneapolis, MN, USA

## Abstract

Age-related macular degeneration (AMD) involves the loss of retinal pigment epithelium (RPE) and photoreceptors and is one of the leading causes of blindness in the elderly. Oxidative damage to proteins, lipids, and DNA has been associated with RPE dysfunction and AMD. In this study, we evaluated oxidative stress in AMD and the efficacy of antioxidant, N-acetyl-L-cysteine (NAC), in protecting RPE from oxidative damage. To test this idea, primary cultures of RPE from human donors with AMD (*n* = 32) or without AMD (No AMD, *n* = 21) were examined for expression of NADPH oxidase (NOX) genes, a source of reactive oxygen species (ROS). Additionally, the cells were pretreated with NAC for 2 hours and then treated with either hydrogen peroxide (H_2_O_2_) or *tert*-butyl hydroperoxide (*t*-BHP) to induce cellular oxidation. Twenty-four hours after treatment, ROS production, cell survival, the content of glutathione (GSH) and adenosine triphosphate (ATP), and cellular bioenergetics were measured. We found increased expression of p22phox, a NOX regulator, in AMD cells compared to No AMD cells (*p* = 0.02). In both AMD and No AMD cells, NAC pretreatment reduced *t*-BHP-induced ROS production and protected from H_2_O_2_-induced cell death and ATP depletion. In the absence of oxidation, NAC treatment improved mitochondrial function in both groups (*p* < 0.01). Conversely, the protective response exhibited by NAC was disease-dependent for some parameters. In the absence of oxidation, NAC significantly reduced ROS production (*p* < 0.001) and increased GSH content (*p* = 0.02) only in RPE from AMD donors. Additionally, NAC-mediated protection from H_2_O_2_-induced GSH depletion (*p* = 0.04) and mitochondrial dysfunction (*p* < 0.05) was more pronounced in AMD cells compared with No AMD cells. These results demonstrate the therapeutic benefit of NAC by mitigating oxidative damage in RPE. Additionally, the favorable outcomes observed for AMD RPE support NAC's relevance and the potential therapeutic value in treating AMD.

## 1. Introduction

Age-related macular degeneration (AMD) is the leading cause of progressive and irreversible vision loss in the aging population [1]. The macula, a small central area of the retina that deteriorates with AMD, is responsible for high acuity and color vision. Approximately 10% of the AMD patient population has the “wet” form of the disease, which manifests as abnormal growth of blood vessels into the retina from the choriocapillaris, a fenestrated blood vessel network outside the eye [2]. The majority of the AMD patient population has “dry” AMD, characterized by the loss of retinal pigment epithelium (RPE) and photoreceptors in the absence of abnormal blood vessel growth. In the last decade, the treatment of wet AMD has significantly improved with the introduction of anti-VEGF therapy [3]. Several new therapeutic strategies against dry AMD have been tested in experimental studies and clinical trials [4], though none has emerged as effective treatments.

The RPE is a single layer of postmitotic pigmented cells located between the photoreceptors and the choriocapillaris. These cells have multiple functions involved in maintaining retinal health including photoreceptor phagocytosis, nutrient transport, and cytokine secretion. Disruption of RPE cell function is a key event in the pathogenesis of AMD [5]. Previous studies suggest that the pathologic mechanism involves mitochondrial dysfunction resulting from oxidative stress and subsequent damage to proteins, lipids, and mtDNA [6–8]. Oxidative stress is a consequence of high levels of reactive oxygen species (ROS) generated physiologically as a by-product of reactions in mitochondria and from several enzymes, including NADPH oxidase (NOX). Thus, strategies that reduce ROS and subsequently oxidative stress may be a potential therapeutic intervention for AMD.

A complication to developing therapeutics is the absence of a defined singular mechanism driving AMD pathology. In addition to age, many risk factors are implicated in the clinical manifestations of AMD, including environmental agents, such as smoking and diet [9] and genetic polymorphisms [10, 11]. However, evidence from numerous studies supports the role of oxidative stress/damage in AMD pathology. For example, human donors with AMD have increased glycation end products and *ω*-(2-carboxyethyl)pyrroles, products of protein oxidation, in their retinas [12]. Additionally, the RPE from AMD donors have elevated levels of antioxidant enzymes, likely a compensatory response to the oxidative environment of the diseased retina [7, 13]. Clinically, the Age-Related Eye Disease Study (AREDS) supports a link between oxidative stress and AMD, showing that supplementation with antioxidants plus zinc slowed progression of the disease [14].

Based on the positive outcomes of the AREDS, antioxidants are an effective approach for protecting the retina of AMD patients. However, the AREDS formulation was effective in ~20% of the patient population with intermediate AMD, thereby providing the rationale for investigating additional antioxidants to treat or prevent AMD progression. N-Acetyl-L-cysteine (NAC) is a sulphur-containing antioxidant that acts as both a free radical scavenger and a precursor of glutathione (GSH), a tripeptide that is an important part of the cellular defense system. To date, NAC has been shown to be an effective antioxidant for eye-related conditions in both mice and humans [15–19]. However, NAC has not been thoroughly investigated as a treatment for dry AMD.

In this study, we used age-matched primary cultures of RPE from human donors with or without AMD to evaluate the efficacy of NAC to improve basal conditions as well as protect cells from an oxidative insult, using either hydrogen peroxide (H_2_O_2_) or tert-butyl hydroperoxide (*t*-BHP). We also analyzed if there was a disease-dependent response to NAC treatment. Our results show that NAC protects against oxidative damage by preventing excessive ROS production, cell death, GSH and ATP depletion, and impairment of mitochondrial oxidative phosphorylation. We also observed that NAC was particularly beneficial for RPE from AMD donors, suggesting relevance to its therapeutic value in treating AMD.

## 2. Materials and Methods

### 2.1. Procurement of Eye Tissue and Grading for AMD

Deidentified donor eyes were obtained from Lions Gift of Sight (formerly known as Minnesota Lions Eye Bank) in Saint Paul, MN. The eyes were obtained with the written consent of the donor or donor's family for use in medical research in accordance with the Declaration of Helsinki. Lions Gift of Sight is licensed by the Eye Bank Association of America (accreditation #0015204) and accredited by the FDA (FDA Established Identifier 3000718538). Donor tissue is exempt from the process of Institutional Review Board approval.

Tissue handling, storage, and donor exclusion criteria are as outlined previously [6, 20]. Evaluation of the presence or absence of AMD was determined by a board-certified ophthalmologist (Sandra R. Montezuma, MD) from stereoscopic fundus photographs of the RPE using the criteria (RPE pigment changes and the presence, size, and location of drusen) established by the Minnesota Grading System (MGS) [13, 21]. Records from Lions Gift of Sight provided demographics (age, gender, cause of death, and time to tissue processing) of the donors used to generate RPE primary cultures (Table 1). See Supplemental Table 1 for information about donors used for each figure.

### 2.2. Cell Culturing

RPE cells were cultured with the conditions previously described [7]. In brief, RPE cells were isolated from human donor eyecups by gently dislodging cells from Bruch's membrane following incubation (15 min) with 0.125% trypsin preheated to 37°C. Cells were grown in Primaria T25 flasks (Corning, Corning, NY) and cultured in MEM alpha medium (Sigma-Aldrich, St. Louis, MO) supplemented with 5% fetal bovine serum (Atlanta Biologicals, Flowery Branch, GA), 1 mM sodium pyruvate, 1% nonessential amino acids, 50 U/mL penicillin, and 50 *μ*g/mL streptomycin. When cells reached confluence (around 1 month), they were passaged using trypsin and split from 1 to 2 (cells from one T25 flask were split into two T25 flasks). Cells in passage 2 or 3 were used for analysis. The cell number and condition are specified under each experimental protocol. Cell samples were selected for the various assays based on their availability. NAC and H_2_O_2_, obtained from Sigma, were added to the RPE cell cultures under the experimental conditions indicated. The concentrations of H_2_O_2_, which gave 25-50% cell death, were chosen based on data from previous studies [7, 22].

### 2.3. Measurement of Cell Death

RPE cells were seeded (5 × 10^3^ cells/well) in black-walled clear-bottom 96-well plates and grown for 48 hr in RPE media containing 1% FBS and no sodium pyruvate. In preliminary experiments to determine the optimal NAC concentration, RPE cells were incubated with NAC (100 to 1000 *μ*M) for 24 hours prior to analysis of cell viability. In subsequent experiments, RPE cells were incubated with NAC (500 *μ*M) for 2 hours and then exposed to different concentrations of H_2_O_2_ (150, 200, and 250 *μ*M) for 24 hr. Cell viability was determined using the CyQUANT Direct Cell Proliferation Assay Kit (Thermo Fisher, Waltham, MA) and Alamar Blue Cell Viability Reagent (Thermo Fisher) according to the instruction of each manufacturer. The fluorescence value for the no treatment control group was considered 100% viable cells. The fluorescence value for cells treated with a lysis buffer was considered 0% viable cells. Fluorescence was determined using a Synergy 2 microplate reader (BioTek, Winooski, VT).

### 2.4. Measurement of ATP Content

RPE cells were seeded (5 × 10^3^ cells/well) in all white 96-well plates and grown for 48 hr in RPE media containing 1% FBS and no sodium pyruvate. In preliminary experiments to determine the optimal NAC concentration, RPE cells were incubated with NAC (100 to 1000 *μ*M) for 24 hours and the content of ATP was determined. In subsequent experiments, RPE cells were incubated with NAC (500 *μ*M) for 2 hours and then exposed to different concentrations of H_2_O_2_ (150, 200, and 250 *μ*M) for 24 hr. The cellular adenosine triphosphate (ATP) production was assayed by using no phenol red DMEM containing 1% FBS and following the manufacturer's protocol for the ATPlite—luminescence ATP detection assay system (PerkinElmer, Waltham, MA). ATP content was estimated from the luminescence of treated cells relative to luminescence of untreated control cells. Values were normalized to the number of viable cells. Luminescence was detected using a microplate reader (BioTek, Synergy 2).

### 2.5. Glutathione (GSH) Analysis

RPE cells were seeded (5 × 10^3^ cells/well) in all white 96-well plates and grown for 48 hr in RPE media containing 1% FBS and no sodium pyruvate. RPE cells were incubated with NAC (500 *μ*M) for 2 hours and then exposed to different concentrations of H_2_O_2_ (150, 200, and 250 *μ*M) for 24 hr. Intracellular GSH levels were measured in media containing no phenol red DMEM and 1% FBS. The protocol followed the manufacturer's instructions for the GSH-Glo Glutathione Assay kit (Promega, Madison, WI). GSH content was estimated from the change in luminescence relative to no treatment controls and then normalized to the number of viable cells. Luminescence was measured on a microplate reader (BioTek, Synergy 2).

### 2.6. Measurement of ROS Formation

RPE cells were seeded (2 × 10^4^ cells/well) in black/clear-bottom 96-well plates and grown for 48 hr in RPE media containing 1% FBS, no sodium pyruvate. The formation of intracellular ROS was assessed using the 2′,7′-dichlorofluorescein diacetate (DCFDA) cellular ROS detection assay kit (abcam, Cambridge, MA) following the manufacturer's protocol. Cells were incubated with DCFDA (25 *μ*M) for 45 min, washed once with fresh media, and incubated with NAC (500 *μ*M) for 1 hour. Subsequently, cells were incubated with 75 *μ*M *t*-BHP for 3 hours. ROS content was calculated based on the fluorescence of treated cells relative to the fluorescence of untreated cells. Fluorescence was read on a microplate reader (BioTek, Synergy 2).

### 2.7. RNA Isolation and qRT-PCR

Total RNA was prepared with the RNeasy Micro Kit (QIAGEN). RNA (300 ng) was used to synthesize cDNA with the SuperScript III First-Strand Synthesis System (Thermo Fisher). To determine the concentration of cDNA, alkaline hydrolysis was performed, and the RiboGreen™ Assay Kit (Thermo Fisher) was used. For alkaline hydrolysis, a mixture of 7 *μ*L cDNA, 2 *μ*L·5 mM EDTA, and 1 *μ*L·1 M NaOH was incubated at 70°C for 20 min; then, 3 *μ*L of 0.5 M Tris-Cl pH 6.4 was added to the mixture. The RiboGreen™ Assay was run using the hydrolyzed samples to quantify the cDNA. The expression of NOX genes was determined using quantitative reverse transcription PCR (qRT-PCR) using a Bio-Rad iQ5 multicolor real-time PCR detection system. Triplicate wells of 25 *μ*L reactions contained 1 ng cDNA, 0.2 *μ*M forward and reverse primers, and 13.5 *μ*L Bio-Rad iQ SYBR Green Supermix. For quantitative detection of *NOX* mRNAs, the following primers were used: *NOX2*, forward 5′-AAGATGCGTGGAAACTACCTAAGAT-3′ and reverse 5′-TCCCTGCTCCCACTAACATCA-3′; *p22phox*, forward 5′-TACTATGTTCGGGCCGTCCT-3′ and reverse 5′-CACAGCCGCCAGTAGGTA-3′; *NOX4*, forward 5′-TATCCAGTCCTTCCGTTGGTT-3′ and reverse 5′-TGAGGTACAGCTGGATGTTGA-3′; and *NOX5*, forward 5′-GCAGGAGAAGATGGGGAGAT-3′ and reverse 5′-CGGAGTCAAATAGGGCAAAG-3′. A standard curve was included with every gene to determine efficiency.

The geometric mean of housekeeping genes, 60S acidic ribosomal protein P0 (ARBP) and hypoxanthine phosphoribosyltransferase 1 (HPRT1), was used to calculate ΔCt of each gene of interest. To determine fold change relative to No AMD, ΔΔCt of each AMD donor was calculated by subtracting the mean ΔCt of No AMD cells. A modified Livak method was used to calculate relative expression using the efficiency for each primer.

### 2.8. Measurement of Bioenergetics

Analysis of bioenergetics was performed on live cells using an XFe^96^ Extracellular Flux Analyzer (Agilent Tech). The analyzer allows real-time measurements of the oxygen consumption rate (OCR) and extracellular acidification rate (ECAR) which are indicators of mitochondrial respiration and glycolysis activity of cells, respectively. Briefly, RPE were plated in 1% serum-containing RPE media without sodium pyruvate and were seeded (4 × 10^4^ cells/well) on XF96 cell culture microplates, which were coated with Cell-Tak (Corning). The following day, RPE cells were pretreated with or without NAC (500 *μ*M) for 2 hr and treated with H_2_O_2_ (500 *μ*M) for 24 hr. The Cell Mito Stress Test or Glycolysis Stress Test assay protocol was performed as detailed by the manufacturer (Agilent Tech) and our previous analysis [7].

### 2.9. Statistical Analysis

**Figure 1:**
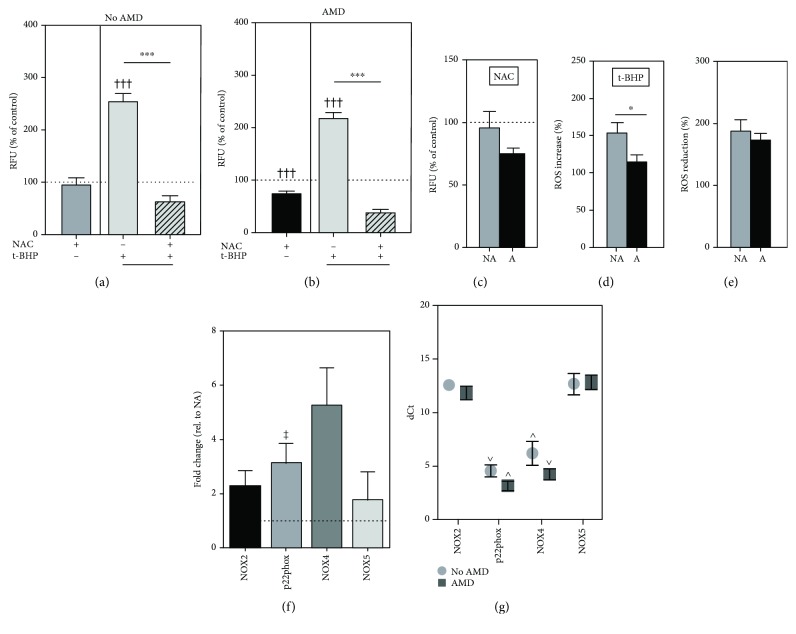
NAC protects against *t*-BHP-induced reactive oxygen species (ROS) production in RPE cells. RPE cells were treated with *t*-BHP (75 *μ*M) for 3 hours with or without NAC (500 *μ*M) pretreatment for 1 hour. The amount of ROS in (a) No AMD (*n* = 7) cells and (b) AMD (*n* = 7) cells was calculated relative to no treatment controls (dotted line). (c) ROS content after NAC treatment was compared between No AMD and AMD cells. (d) Percent increase (*t*-BHP—no treatment) of ROS in *t*-BHP-treated cells and (e) percent reduction (*t*-BHP—NAC+*t*-BHP) of ROS in NAC-pretreated cells were compared between No AMD and AMD donors. NA: No AMD; A: AMD. (f) mRNA expression of NOX family genes in No AMD (*n* = 7) and AMD (*n* = 8) cells was measured by real-time PCR. Results are fold change in expression relative to the average for No AMD samples (dotted line). (g) Expression of NOX family genes relative to housekeeping genes (dCt). One-sample *t*-tests were used to compare NAC treatment or *t*-BHP alone to no treatment in No AMD and AMD groups (a, b). Unpaired *t*-tests were used to compare *t*-BHP treatment to NAC + *t*-BHP treatment in (a, b). Unpaired *t*-tests were used to compare responses of No AMD and AMD in (c–e). Unpaired *t*-tests were used to compare basal expression of NOX genes in (f). One-way ANOVA with Tukey's multiple comparison test was used to compare dCt values of NOX genes in (g). Data are the mean (±SEM). ^†^ denotes significance from the no treatment control, and ^∗^ denotes significance between conditions. ^∗^ *p* < 0.05 and ^∗∗∗^ or ^†††^ *p* < 0.001 were statistically significant. ^ǂ^ denotes significance in relative expression of NOX genes between No AMD and AMD groups. ^∧^ and ^∨^ denote significance between dCt values of NOX genes within No AMD or AMD groups.

**Figure 2:**
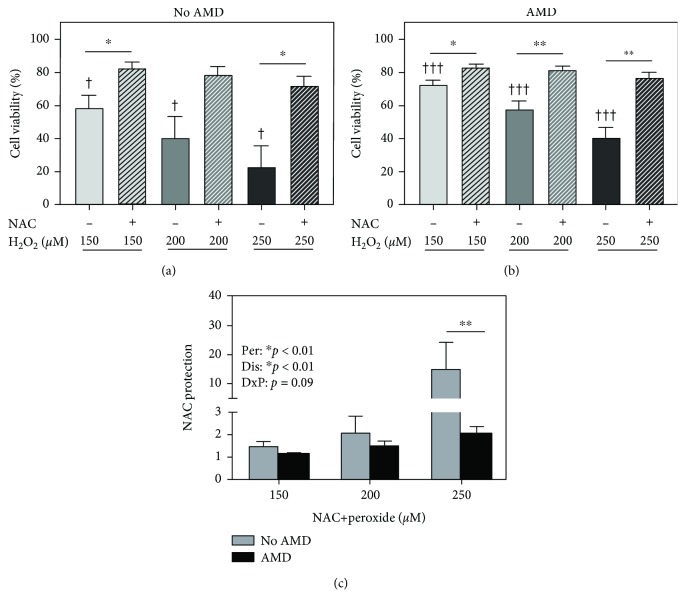
NAC protects against H_2_O_2_-induced cell death. RPE cells were treated with H_2_O_2_ (150, 200, and 250 *μ*M) for 24 hours with or without NAC (500 *μ*M) pretreatment for 2 hours. Cell viability in (a) No AMD (*n* = 5) cells and (b) AMD (*n* = 10) cells was calculated relative to the no treatment control. (c) NAC protection was calculated as NAC+H_2_O_2_ relative to H_2_O_2_ alone. One-sample *t*-tests were used to compare H_2_O_2_ treatment to no treatment in No AMD and AMD groups (a, b). Unpaired *t*-tests were used to compare peroxide to NAC+peroxide treatments in (a, b). Two-way ANOVA with Sidak's post hoc was used to compare the effect of the disease state (No AMD vs. AMD) and H_2_O_2_ concentration in (c). Per: peroxide effect; Dis: disease effect; DxP: interaction between disease effect and peroxide effect. Data are the mean (±SEM). ^†^ denotes significance from the no treatment control, and ^∗^ denotes significance between conditions. ^∗^ or ^†^ *p* < 0.05, ^∗∗^ *p* < 0.01, and ^†††^ *p* < 0.001 were statistically significant.

**Figure 3:**
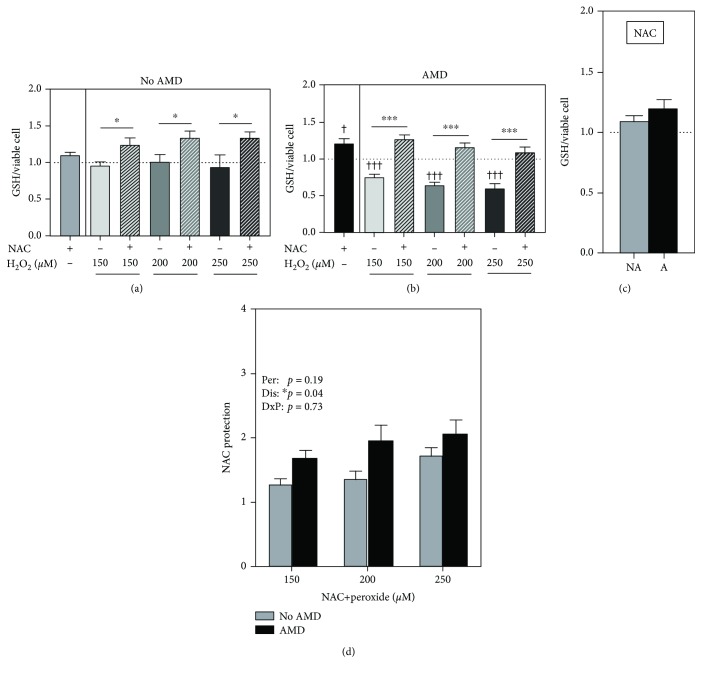
NAC protects against GSH depletion. RPE cells were treated with H_2_O_2_ (150, 200, and 250 *μ*M) for 24 hours with or without NAC (500 *μ*M) pretreatment for 2 hours. GSH levels in (a) No AMD (*n* = 6) cells and (b) AMD (*n* = 15) cells were calculated relative to the no treatment control (dotted line). (c) GSH content after NAC treatment was compared between No AMD (NA) cells and AMD (A) cells. (d) NAC protection was calculated as NAC+H_2_O_2_ relative to H_2_O_2_ alone. One-sample *t*-tests were used to compare NAC treatment or H_2_O_2_ alone to no treatment in No AMD and AMD groups (a, b). Unpaired *t*-tests were used to compare H_2_O_2_ to NAC+H_2_O_2_ treatments in (a, b). An unpaired *t*-test was used to compare GSH content in NAC-treated No AMD cells to NAC-treated AMD cells in (c). Two-way ANOVA with Sidak's post hoc was used to compare the effect of the disease state (No AMD vs. AMD) and H_2_O_2_ concentration in (d). Per: peroxide effect; Dis: disease effect; DxP: interaction between disease effect and peroxide effect. Data are the mean (±SEM). ^†^ denotes significance from the no treatment control, and ^∗^ denotes significance between conditions. ^∗^ *p* < 0.05 and ^∗∗∗^ or ^†††^ *p* < 0.001 were statistically significant.

**Figure 4:**
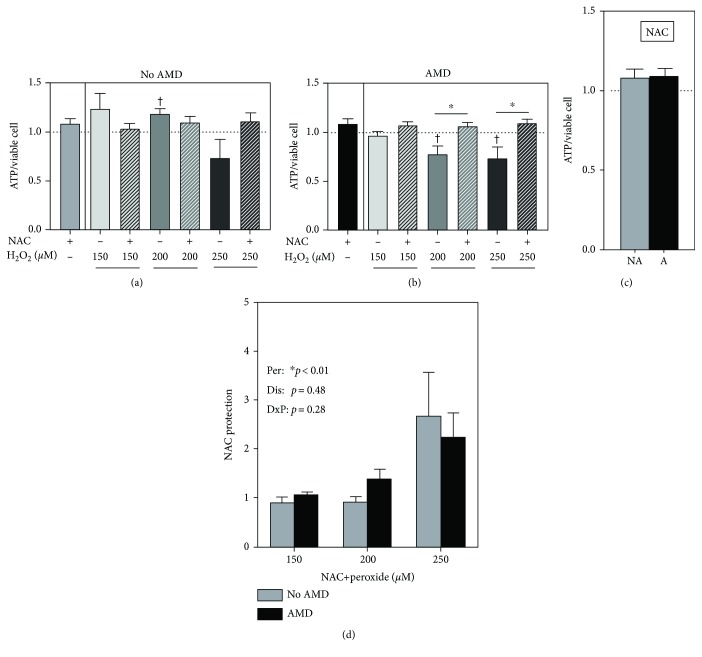
NAC protects against H_2_O_2_-induced ATP depletion. RPE cells were treated with H_2_O_2_ (150, 200, and 250 *μ*M) for 24 hours with or without NAC (500 *μ*M) pretreatment for 2 hours. ATP content in (a) No AMD (*n* = 6) cells and (b) AMD (*n* = 12) cells was calculated relative to the no treatment control. (c) ATP content after NAC treatment was compared between No AMD (NA) and AMD (A) cells. (d) NAC rescue was calculated as NAC+H_2_O_2_ relative to H_2_O_2_ alone. One-sample *t*-tests were used to compare NAC treatment or H_2_O_2_ alone to no treatment in No AMD and AMD groups (a, b). Unpaired *t*-tests were used to compare H_2_O_2_ to NAC+H_2_O_2_ treatments in (a, b). An unpaired *t*-test was used to compare ATP content in NAC-treated No AMD cells to NAC-treated AMD cells in (c). Two-way ANOVA with Sidak's post hoc was used to compare the effect of the disease state (No AMD vs. AMD) and H_2_O_2_ concentration in (d). Per: peroxide effect; Dis: disease effect; DxP: interaction between disease effect and peroxide effect. Data are the mean (±SEM). ^†^ denotes significance from the no treatment control, and ^∗^ denotes significance between conditions. ^∗^ or ^†^ *p* < 0.05 was statistically significant.

Prior to statistical analysis, Grubb's test was performed on each dataset to remove the single largest outlier. All treatment data were normalized to no treatment condition for each donor (fold change relative to no treatment). Statistical analysis was performed on log transformed fold change values. One-sample *t*-tests, with a hypothetical value of zero, were used to compare NAC treatment or peroxide treatment alone to no treatment control data. Unpaired *t*-tests were used to compare peroxide treatment to NAC+peroxide treatment data. Unpaired *t*-tests were also used to compare NAC treatment response in No AMD to AMD cells. Two-way ANOVA with Sidak's multiple comparison was used to compare the effect of the disease state (No AMD vs. AMD) and hydrogen peroxide concentrations on data in Figures 2(c), 3(d), and 4(d). Unpaired *t*-tests of the ΔΔCt values were used to compare NOX gene expression levels between No AMD and AMD cells in Figure 1(f). One-way ANOVA with Tukey's post hoc was used to compare ΔCt values between NOX genes in Figure 1(g). Data was analyzed using statistical software in GraphPad Prism 7 (GraphPad, La Jolla, CA). *p* ≤ 0.05 was considered statistically significant. All results are presented as the mean ± SEM.

## 3. Results

### 3.1. Background

Clinical information and demographics of the donors used in this study are provided in Table 1. RPE cultures were obtained from donors without AMD (No AMD, *n* = 21; aged 49-77) and donors with AMD (AMD, *n* = 32; aged 49-89). The average age of donors with AMD (71.8 ± 11.8; mean ± SD) was 10% older than that of No AMD donors (64.0 ± 9.8) (*p* = 0.01), which is consistent with the high prevalence of this disease in individuals over 65 years [1]. The average time from death to RPE cell harvesting was not different for No AMD (18.2 ± 4.3 hours) and AMD (19.8 ± 4.0 hours) donors (*p* = 0.12).

RPE from several donors without AMD (*n* = 3) were used to determine the optimal NAC concentration. In preliminary experiments, a range of concentrations (100 to 1000 *μ*M) was used to determine the effect on cell viability and ATP. Data were normalized to no treatment controls. Using two different cell viability assays, there was no change in cell survival at all concentrations of NAC. However, there was an ~20% increase in ATP content at 500 *μ*M (Supplemental Figure 1). At the highest dose of NAC (1000 *μ*M), ATP content decreased by 30% (*p* = 0.03) signifying that this dose was outside the optimal range for our experimental system. Based on these experimental results, 500 *μ*M NAC was chosen for subsequent experiments. This dose is also consistent with a previous study reporting that 500 *μ*M NAC was found in the serum of patients after intravenous injection of NAC [23].

### 3.2. NAC Attenuates Intracellular ROS Levels

To investigate the antioxidant effect of NAC on RPE cells, intracellular reactive oxygen species (ROS) levels were measured before and after exposure to *t*-BHP. We found that treatment with NAC (500 *μ*M) alone had no effect on ROS levels in No AMD cells (Figure 1(a)) but caused a significant decrease in basal ROS content (25%) in AMD (*p* = 0.0009) cells compared to no treatment controls (Figure 1(b)). However, ROS content was not significantly different with NAC treatment when comparing cells from donors with and without AMD (Figure 1(c)). While exposure to *t*-BHP significantly increased the amount of ROS in both groups (*p* < 0.001 for No AMD and AMD, Figures 1(a) and 1(b)), the increase was significantly more (*p* = 0.04) in No AMD (155%) cells compared to AMD (118%) cells (Figure 1(d)). Pretreatment with NAC prior to *t*-BHP exposure significantly reduced the levels of ROS in both No AMD (*p* < 0.0001) and AMD (*p* < 0.0001) cells (Figures 1(a) and 1(b)). ROS was reduced to a similar extent in both groups of cells (Figure 1(e)). Our results suggest that NAC can suppress ROS production in RPE under conditions of cellular oxidation.

### 3.3. Increased Expression of NOX Family Genes in AMD Cells

Evidence suggests that NOX plays an important role in ROS generation and redox signaling pathways in RPE [24]. Expression of NOX family genes, NOX2, p22phox, NOX4, and NOX5, was measured under basal conditions in cells from donors with or without AMD. When comparing expression in AMD cells to that in No AMD cells, p22phox was significantly higher (*p* = 0.02) (Figure 1(f)). NOX2, NOX4, and NOX5 were consistently higher in AMD cells; however, the difference did not reach statistical significance. Utilizing dCt to compare mRNA levels (lower dCt indicates higher expression), we found that expression of NOX4 and p22phox was significantly more abundant than that of NOX2 and NOX5 in RPE from both No AMD and AMD donors (Figure 1(g)). These results suggest that NOX may contribute to ROS levels in RPE cells, especially in RPE from donors with AMD.

### 3.4. NAC Prevents Oxidative Stress-Induced Cytotoxicity

To determine if NAC could protect against oxidation-induced cell death, RPE were exposed to increasing amounts of H_2_O_2_. In RPE from donors with and without AMD, H_2_O_2_ significantly decreased cell viability in a dose-dependent manner (Figure 2(a), *p* < 0.05; Figure 2(b), *p* < 0.001). At all three concentrations of H_2_O_2_, pretreatment with NAC significantly protected against cell death in RPE from both No AMD (*p* = 0.03 at 150 *μ*M and 250 *μ*M, Figure 2(a)) and AMD (*p* = 0.01 at 150 *μ*M, *p* = 0.002 at 200 *μ*M, and *p* = 0.004 at 250 *μ*M, Figure 2(b)) donors. Of note, there was a dose-dependent increase in NAC protection of cell viability, and the overall effect was significantly better for No AMD cells, particularly at 250 *μ*M H_2_O_2_ (*p* = 0.006, Figure 2(c)). However, this higher protective effect of NAC is due to the decreased viability of cells from donors without AMD following H_2_O_2_ treatment compared to AMD cells, where more modest cell death was observed. These results demonstrate that NAC is able to protect RPE cells when exposed to oxidative stress that would otherwise induce 25% to 80% cell death.

### 3.5. NAC Protects against GSH Depletion

NAC can serve either as a direct antioxidant via its reactive sulfhydryl or as a precursor for synthesis of GSH. To gain mechanistic insight into how NAC protects against ROS-induced cell death, we assessed whether NAC preserves thiol content in our RPE cells. The intracellular GSH level was measured after treatment with NAC before and after exposure to H_2_O_2_. Treatment with NAC alone caused a 10% increase in GSH content in No AMD (*p* = 0.06, Figure 3(a)) and a significant 20% increase in GSH content in AMD (*p* = 0.02, Figure 3(b)) cells compared to untreated controls. However, the extent of GSH replenishment by NAC was not significantly different between the two groups (Figure 3(c)). Upon exposure to increasing levels of H_2_O_2_, GSH content did not change in cells from donors without AMD (Figure 3(a)). In contrast, there was a significant dose-dependent decrease in GSH content (25% to 40%) induced by H_2_O_2_ in AMD cells (Figure 3(b)). Pretreatment with NAC prevented GSH depletion in RPE from both No AMD (Figure 3(a)) and AMD (Figure 3(b)) donors at all concentrations of H_2_O_2_. The protective effect of NAC on GSH was significantly greater in RPE from AMD donors compared to RPE from No AMD donors (*p* = 0.04, Figure 3(d)). These results demonstrate the protective action of NAC in preventing the depletion of GSH in RPE cells treated with H_2_O_2_.

### 3.6. NAC Prevents ATP Depletion

A previous work has shown that NAC pretreatment protects RPE from mitochondrial dysfunction and ATP reduction following exposure to the oxidizing conditions of high glucose [25]. To determine if NAC has an effect on cell bioenergetics, we measured ATP content before and after H_2_O_2_ treatment. Treatment with NAC alone did not significantly change ATP content in cells from either No AMD (Figure 4(a)) or AMD (Figure 4(b)) donors and was not different between groups (Figure 4(c)). In RPE from No AMD donors, H_2_O_2_ and NAC had no effect on ATP (Figure 4(a)). The effect of H_2_O_2_ was more dramatic in AMD donors; ATP content decreased by 23% and 30% at 200 *μ*M (*p* = 0.03) and 250 *μ*M (*p* = 0.03), respectively (Figure 4(b)). NAC completely prevented H_2_O_2_-induced ATP depletion at 200 *μ*M (*p* = 0.01) and 250 *μ*M (*p* = 0.01, Figure 4(b)). The extent of NAC protection on ATP depletion was similar in both No AMD and AMD cells (*p* = 0.48, Figure 4(d)). These results show that NAC is able to protect against ATP depletion caused by H_2_O_2_ treatment in RPE cells.

### 3.7. NAC Protects against Mitochondrial Dysfunction but Not Glycolysis

**Figure 5:**
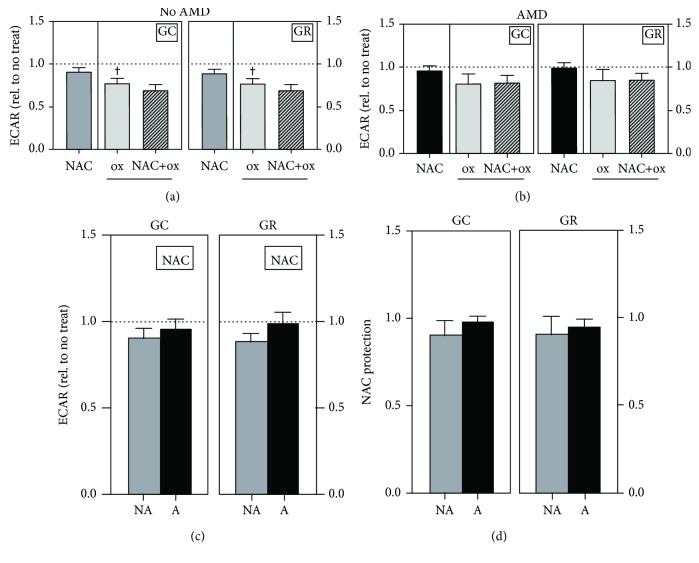
NAC treatment does not affect glycolysis. RPE cells were treated with H_2_O_2_ (500 *μ*M) for 24 hours with or without NAC (500 *μ*M) pretreatment for 2 hours. Relative extracellular acidification rate (ECAR) levels in No AMD (*n* = 6) and AMD (*n* = 7) cells were normalized to no treatment for each donor. Glycolytic capacity (GC) and glycolytic reserve (GR) were calculated for (a) No AMD and (b) AMD cells. (c) Relative ECAR after NAC treatment was compared between No AMD (NA) and AMD (A) cells. (d) NAC protection was calculated as NAC+H_2_O_2_ (NAC+ox) relative to H_2_O_2_ (ox) alone. All data are the mean (±SEM). One-sample *t*-tests were used to compare NAC treatment or H_2_O_2_ alone to no treatment in No AMD and AMD groups (a, b). Unpaired *t*-tests were used to compare H_2_O_2_ treatment to NAC+H_2_O_2_ treatment in (a, b). Unpaired *t*-tests were used to compare responses of No AMD to AMD in (c, d). Data are the mean (±SEM). ^†^ denotes significance from no treatment control. ^†^ *p* < 0.05 was considered statistically significant.

To provide a more comprehensive evaluation of the source of improved ATP content, we examined both glycolysis and mitochondrial oxidative phosphorylation, two energy pathways that produce ATP, using an extracellular flux analyzer. In measuring glycolytic function, treatment with NAC alone had no effect on glycolytic capacity or glycolytic reserve in both No AMD (Figure 5(a)) and AMD (Figure 5(b)) cells and there was no difference in their response to NAC (Figure 5(c)). Further, in No AMD cells, H_2_O_2_ significantly decreased glycolytic capacity (~20%, *p* = 0.02) and glycolytic reserve (~20%, *p* = 0.02) compared to no treatment controls (Figure 5(a)). However, H_2_O_2_ did not significantly decrease glycolytic capacity or reserve in AMD cells (Figure 5(b)). NAC pretreatment prior to oxidation did not improve glycolytic capacity or glycolytic reserve in either group (Figures 5(a), 5(b), and 5(d)). These results suggest that No AMD RPE are more sensitive to H_2_O_2_-induced reduction in glycolysis and that NAC had no effect on either basal glycolysis or protection from H_2_O_2_-induced glycolytic inactivation.

**Figure 6:**
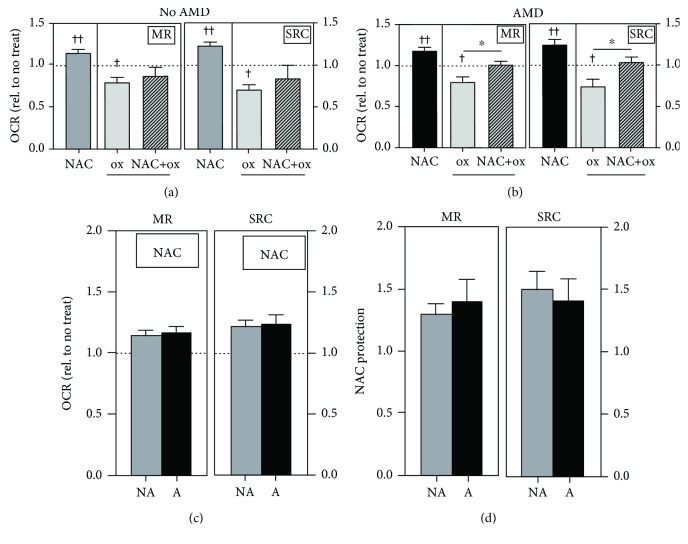
NAC protects against H_2_O_2_-induced mitochondrial oxidative phosphorylation impairment. RPE cells were treated with H_2_O_2_ (500 *μ*M) for 24 hours with or without NAC (500 *μ*M) pretreatment for 2 hours. Relative oxygen consumption rate (OCR) levels in No AMD (*n* = 6) and AMD (*n* = 11) cells were normalized to no treatment for each donor. Maximal respiration (MR) and spare respiratory capacity (SRC) were calculated for (a) No AMD and (b) AMD cells. (c) Relative OCR after NAC treatment was compared between No AMD (NA) and AMD (A) cells. (d) NAC protection was calculated as NAC+H_2_O_2_ (NAC+ox) relative to H_2_O_2_ (ox) alone. One-sample *t*-tests were used to compare NAC treatment or H_2_O_2_ alone to no treatment (set to 1) in No AMD and AMD groups (a, b). Unpaired *t*-tests were used to compare H_2_O_2_ treatment to NAC+H_2_O_2_ treatment in (a, b). Unpaired *t*-tests were used to compare responses of No AMD to AMD in (c, d). Data are the mean (±SEM). ^†^ denotes significance from the no treatment control, and ^∗^ denotes significance between conditions. ^∗^ or ^†^ *p* < 0.05 and ^††^ *p* < 0.01 were statistically significant.

To investigate if NAC preservation of ATP content after H_2_O_2_ exposure was due to protection of mitochondrial oxidative phosphorylation, OCR was measured using the Cell Mito Stress Test. Traces of average OCR (normalized to baseline) are shown in Supplemental Figure 2. Compared to no treatment, NAC-treated cells exhibited a significant increase in maximal respiration (16%) and spare respiratory capacity (25%) in No AMD (*p* < 0.01, Figure 6(a)) and AMD (*p* < 0.01, Figure 6(b)) cells. This improvement in mitochondrial function was not different between groups (Figure 6(c)). Exposure to H_2_O_2_ significantly decreased maximal respiration (20%) and spare respiratory capacity (30%) in No AMD cells and significantly reduced maximal respiration (20%) and spare respiratory capacity (25%) in AMD cells compared to no treatment controls (Figures 6(a) and 6(b)). NAC pretreatment had no effect on cells from donors with No AMD (Figure 6(a)) but significantly improved maximal respiration (*p* = 0.02) and spare respiratory capacity (*p* = 0.05) in AMD cells compared to cells treated with H_2_O_2_ alone (Figure 6(b)). NAC's protection of maximal respiration or spare respiratory capacity was not different between groups (Figure 6(d)). These results suggest that NAC's ability to maintain mitochondrial oxidative phosphorylation can partially explain the preservation of ATP content under conditions of cellular oxidation.

## 4. Discussion

In this study, we examined the effects of NAC treatment on primary cultures of age-matched human RPE from donors graded for the presence (AMD) or absence (No AMD) of AMD. We demonstrated that NAC protects against oxidative stress by blocking excessive ROS accumulation (Figure 1) and preventing both H_2_O_2_-induced cell death (Figure 2) and GSH depletion (Figure 3) in RPE from both No AMD and AMD donors. NAC also improved basal mitochondrial function (Figure 6) in both groups of cells. Several beneficial effects specific for AMD RPE were observed, including a reduction in basal ROS (Figure 1), an increase in basal GSH content, and greater NAC protection after oxidation (Figure 3). These results demonstrate NAC's positive effect on protecting RPE from an oxidative insult. Additionally, the favorable outcomes observed for RPE from AMD donors support NAC's relevance and potential therapeutic value in treating AMD.

Primary cultures of RPE from AMD donors have been a valuable model system for studying the disease mechanism [7] and testing drug efficacy. A limitation of our model system is that cells in culture do not fully replicate the retinal environment. Additionally, due to the nature of procuring donor tissue, the distribution of males and females was not always balanced in each assay, which may have influenced our results. On the other hand, one of the greatest strengths of this study is the large number of individual donors tested. Publications that use far fewer individual donors could potentially bias their results. While there are caveats, this model system provides a unique opportunity to replicate aspects of the disease that are essential for developing therapies to treat AMD.

Oxidation-induced RPE cell death has previously been suggested as the critical pathologic event in AMD [5, 26]. Several unique circumstances put RPE at greater risk for oxidative damage relative to most other cell types. RPE are adjacent to the choriocapillaris, the main source of oxygen for the outer retina, placing them in a highly oxidative environment [26]. Also contributing to the oxidizing environment within the RPE are the ROS generated as a product of the reaction of light with abundant photosensitizers, such as lipofuscin and melanin. The daily phagocytosis of photoreceptor outer segments containing easily oxidized polyunsaturated fatty acids also generates ROS within the RPE. Thus, it is essential that RPE have multiple systems in place for protecting against ROS-induced damage. When oxidative damage reaches a critical threshold, RPE cell death ensues [27–29]. The RPE cell death that occurs with AMD supports the notion that therapies designed to reduce ROS may be a viable option to treat AMD.

Mitochondria are a major source of ROS, not only in RPE but also in all eukaryotic cells. These ROS are generated as a by-product of the reduction of oxygen during oxidative phosphorylation [30]. Mitochondrial ROS are kept in check by mitochondria-localized antioxidants, such as MnSOD and GPX. However, under pathological conditions, overproduction of ROS can occur, causing damage to mitochondrial lipids, proteins, and mitochondrial DNA (mtDNA), ultimately leading to a loss in mitochondrial function. Strong experimental evidence supports the idea that mitochondrial damage is one of the key events driving AMD pathology [8]. Multiple studies have reported increased mtDNA damage and reduced mitochondrial function in RPE from human donors with AMD [6, 7, 20, 22, 31]. In the current study, we observed that NAC had a positive effect on basal mitochondrial function for RPE from donors with and without AMD (Figure 6). NAC was also able to maintain mitochondrial function during an oxidative insult in AMD RPE (Figure 6). These results support the therapeutic value of NAC considering the importance of maintaining RPE mitochondrial function for overall retinal health.

ROS can also be produced by several enzyme systems including xanthine oxidase, uncoupled nitric oxide synthase, and NADPH oxidases [32]. NADPH oxidase (NOX) is the most well-known nonmitochondrial source of ROS [33]. To the best of our knowledge, our study was the first to compare NOX expression between RPE cells from No AMD and AMD donors. We found expression of NOX2, NOX4, and p22phox in human primary RPE cells, consistent with findings from other studies using ARPE-19 cells [24, 33–35]. p22phox is a subunit required for activating and regulating NOX2 and NOX4 [36]. Uniquely, we also found that NOX5 is expressed in human primary RPE cells, albeit at low abundance. We found an overall increased expression of NOX family genes in RPE from AMD donors; however, only p22phox expression reached statistical significance. These results suggest that NOX may play a greater role in redox signaling in RPE with AMD. While overabundance of ROS can be detrimental, ROS is necessary for generating a protective response, for example, through Nrf2 activation [26]. Therefore, the upregulation of NOX family members may be a protective response to the diseased environment of the retina.

Under physiological conditions, endogenous antioxidant enzymes and GSH quickly eliminate ROS. Oxidative stress occurs when there is an imbalance between the oxidant and antioxidant systems, allowing for accumulation of excessive ROS [37]. Oxidative stress can evolve into cellular damage when disproportionate levels of ROS overwhelm the endogenous scavengers, which occurs under pathophysiological conditions [37]. Cells defend against ROS by inducing expression of numerous antioxidant enzymes, such as superoxide dismutase (SOD), catalase, and glutathione peroxidase (GPX) [38, 39]. Of note, our lab found that RPE tissue from AMD donors exhibits substantial upregulation of MnSOD and catalase relative to RPE from donors without AMD [13]. Additionally, we found increased GPX expression upon oxidative insult in cultured RPE from donors with AMD but not in age-matched control RPE [7]. These cultured AMD RPE were also more resistant to oxidation, suggesting that the oxidative environment of the diseased retina experienced *in vivo* stimulates the RPE to mount a compensatory response to minimize oxidative damage. A potential mechanism may include the utilization of GSH, a highly abundant tripeptide (consisting of glycine, cysteine, and glutamic acid) responsible for controlling the cellular redox status [40]. In the current study, we observed that NAC treatment significantly reduced basal ROS and increased basal GSH in AMD RPE (Figures 1 and 3). An acute bolus of H_2_O_2_ depleted GSH more extensively in the cells derived from AMD donors (Figure 3). These results suggest that a more oxidative cellular environment is present in AMD RPE. Additionally, protection by GSH may be one of the important pathways utilized by diseased RPE to maintain the redox status and protect from oxidative damage.

Antioxidants provide a promising avenue to alleviate excessive ROS accumulation associated with dry AMD [41]. Also called “free radical scavengers,” antioxidants play a protective role in oxygen-related stress injuries. A prospective cohort study showed that low levels of dietary antioxidants and zinc could be a risk factor for developing AMD [42]. Several studies have shown that antioxidants, such as neuroligin-3, eupatilin, 3H-1,2-dithiole-3-thione, and escin, prevented or decreased oxidative stress in ARPE-19, an immortalized RPE cell line [43–46], providing rationale for continued pursuit of effective antioxidant therapeutics.

The antioxidant used in this study, NAC, has been extensively studied as a therapy for a variety of eye ailments. However, our study is the first to investigate the efficacy of NAC in primary RPE cells from donors with or without AMD. In vitro, NAC increased cell viability in ARPE-19 cells following oxidative stress [47] and promoted DNA synthesis in primary RPE cells exposed to *t*-BHP [48]. In a mouse model of photoinduced retinal degeneration, intraperitoneal injections of NAC suppressed oxidative and ER stresses, while also inhibiting ROS accumulation in the Balb/c mouse retina [15]. In a murine dry eye study, eye drop administration of NAC diminished the levels of ROS and inflammasome signaling [16]. These studies are consistent with our results showing that NAC treatment reduced ROS (Figure 1) and improved cell viability (Figure 2) with application of an oxidizing agent.

NAC has many properties that make it an appealing drug for AMD patients. NAC is commercially available as both an FDA-approved prescription drug and an over-the-counter dietary supplement, thereby facilitating its use in the clinic. It also has a long history of successful use in multiple conditions where elevated ROS induce pathology. Currently, NAC is approved for oral and intravenous administration in the treatment of acetaminophen overdose [49–51]. In clinical trials, NAC has been administered as a dietary supplement to treat psychiatric disorders [52]. In a randomized study with twenty patients, topical administration of NAC was effective in the treatment of Meibomian gland dysfunction, a chronic condition of the eyelids [18]. NAC has been effective in protecting the retina from oxidative damage when applied topically to the eye, as shown in rd10+/+ mice, a model of retinitis pigmentosa [17]. These results have important implications for AMD, because they show that when applied to the cornea, NAC was able to penetrate to the posterior segment and protect the retina.

As a drug, NAC is an ideal xenobiotic due to its ability to directly enter endogenous biochemical processes because of its own metabolism [53]. NAC controls the redox state in the cells by reducing free radicals directly through its scavenging activity, and it reduces oxidized proteins through its thiol-disulfide exchange activity [54, 55]. Indirectly, NAC regulates the redox state through conversion to cysteine, the precursor for GSH, an important component of the antioxidant defense system [49]. Depletion of GSH is a critical signal that regulates the activation of cell death pathways and is one of the markers of oxidative stress [56]. GSH is a potent scavenger of ROS, but its levels decline with aging and AMD [57–59], highlighting the benefit of significant GSH replenishment with NAC treatment reported in this study (Figure 3).

## 5. Conclusions

In summary, our study was the first to investigate the effect of NAC on age-matched primary human RPE cells graded for the presence or absence of AMD. Through its antioxidant properties, NAC protected RPE cells from oxidative damage by preventing ROS accumulation, GSH and ATP depletion, cell death, and mitochondrial dysfunction. Our findings support further evaluation of the pharmacological value of NAC or other thiol-containing compounds in preventing or delaying progression of visual loss in AMD patients.

## Figures and Tables

**Table 1:** Donor characteristics and clinical information^a^.

Disease state^b^	Sample^c^ (*n*)	Sex Male (*n*)	Sex Female (*n*)	Age^d^ (mean ± SD)	Time^e^ (mean ± SD)	Cause of death^f^ (*n*)
No AMD	21	10	11	64 ± 9.8	18 ± 4.2	ABI (2), ACE (2), ALS (1), cancer (5), CVA (2), ESRD (1), MI (1), pneumonia (1), respiratory failure (2), and sepsis (4)
AMD	32	23	9	71 ± 11.7	19 ± 4.0	ACE (3), cancer (6), cardiac arrest (2), CHF (4), COPD (2), CVA (2), ESLD (1), MI (1), multisystem organ failure (1), pneumonia (2), sepsis (7), and unknown (1)

ABI: anoxic brain injury; ACE: acute cardiac event; ALS: amyotrophic lateral sclerosis; CHF: congestive heart failure; CVA: cerebrovascular accident; COPD: chronic obstructive pulmonary disease; ESLD: end-stage liver disease; ESRD: end-stage renal disease; MI: myocardial infarction. ^a^Information supplied by Lions Gift of Sight (St. Paul, MN). ^b^Minnesota Grading System (MGS) was used to evaluate the stage of AMD in eye bank eyes (Olsen and Feng [21]). No AMD: MGS1; AMD: MGS 2, 3, or 4. ^c^Sample number indicates the total donors with or without AMD used in the current study. ^d^Age of donors is significantly different between No AMD and AMD groups (*p* = 0.02) by *t*-test analysis. ^e^The time from death to harvesting in hours. ^f^The number of donors for each cause of death is indicated in parentheses.

## Data Availability

Data used to support the findings in this study are included within this manuscript.

## Abbreviations

AMD: Age-related macular degeneration
ATP: Adenosine triphosphate
GSH: Glutathione
H_2_O_2_: Hydrogen peroxide
mtDNA: Mitochondrial DNA
NAC: N-Acetyl-L-cysteine
NOX: Nicotinamide adenine dinucleotide phosphate oxidase
ROS: Reactive oxygen species
RPE: Retinal pigment epithelium
*t*-BHP: *tert*-Butyl hydroperoxide.

## Supplementary Materials

Supplementary Materials Figure S1: characterization of NAC. Figure S2: traces from Cell Mito Stress Test analysis of the oxygen consumption rate. Supplemental Table 1: donor gender and ages used in each figure.

## References

[1] Wong W. L., Su X., Li X. (2014). Global prevalence of age-related macular degeneration and disease burden projection for 2020 and 2040: a systematic review and meta-analysis. *The Lancet Global Health*.

[2] Qiu F., Matlock G., Chen Q. (2017). Therapeutic effects of PPAR*α* agonist on ocular neovascularization in models recapitulating neovascular age-related macular degeneration. *Investigative Opthalmology & Visual Science*.

[3] Mehta H., Tufail A., Daien V. (2018). Real-world outcomes in patients with neovascular age-related macular degeneration treated with intravitreal vascular endothelial growth factor inhibitors. *Progress in Retinal and Eye Research*.

[4] Clinicaltrials.Gov/ct2/Results?Cond=Age-Related+Macular+Degeneration

[5] Datta S., Cano M., Ebrahimi K., Wang L., Handa J. T. (2017). The impact of oxidative stress and inflammation on RPE degeneration in non-neovascular AMD. *Progress in Retinal and Eye Research*.

[6] Terluk M. R., Kapphahn R. J., Soukup L. M. (2015). Investigating mitochondria as a target for treating age-related macular degeneration. *Journal of Neuroscience*.

[7] Ferrington D. A., Ebeling M. C., Kapphahn R. J. (2017). Altered bioenergetics and enhanced resistance to oxidative stress in human retinal pigment epithelial cells from donors with age-related macular degeneration. *Redox Biology*.

[8] Fisher C. R., Ferrington D. A. (2018). Perspective on AMD pathobiology: a bioenergetic crisis in the RPE. *Investigative Ophthalmology & Visual Science*.

[9] Buschini E., Zola M., FEA A. M. (2015). Recent developments in the management of dry age-related macular degeneration. *Clinical Ophthalmology*.

[10] Nowak J. Z. (2006). Age-related macular degeneration (AMD): pathogenesis and therapy. *Pharmacological Reports*.

[11] Mousavi M., Armstrong R. A. (2013). Genetic risk factors and age-related macular degeneration (AMD). *Journal of Optometry*.

[12] Gu J., Pauer G. J. T., Yue X., Anderson R., Hollyfield J., Lavail M. (2010). Proteomic and genomic biomarkers for age-related macular degeneration. *Retinal Degenerative Diseases*.

[13] Decanini A., Nordgaard C. L., Feng X., Ferrington D. A., Olsen T. W. (2007). Changes in select redox proteins of the retinal pigment epithelium in age-related macular degeneration. *American Journal of Ophthalmology*.

[14] Age-Related Eye Disease Study Research Group (2001). A randomized, placebo-controlled, clinical trial of high-dose supplementation with vitamins C and E, beta carotene, and zinc for age-related macular degeneration and vision loss: AREDS report no. 8. *Archives of Ophthalmology*.

[15] Osada H., Okamoto T., Kawashima H. (2017). Neuroprotective effect of bilberry extract in a murine model of photo-stressed retina. *PLoS One*.

[16] Zheng Q., Ren Y., Reinach P. S. (2014). Reactive oxygen species activated NLRP3 inflammasomes prime environment-induced murine dry eye. *Experimental Eye Research*.

[17] Lee S. Y., Usui S., Zafar A. B. (2011). N-Acetylcysteine promotes long-term survival of cones in a model of retinitis pigmentosa. *Journal of Cellular Physiology*.

[18] Akyol-Salman Í., Azizi S., Mumcu U., Baykal O. (2010). Efficacy of topical *N*-acetylcysteine in the treatment of meibomian gland dysfunction. *Journal of Ocular Pharmacology and Therapeutics*.

[19] Majid A. S. A., Yin Z. Q., Ji D. (2013). Sulphur antioxidants inhibit oxidative stress induced retinal ganglion cell death by scavenging reactive oxygen species but influence nuclear factor (erythroid-derived 2)-like 2 signalling pathway differently. *Biological and Pharmaceutical Bulletin*.

[20] Karunadharma P. P., Nordgaard C. L., Olsen T. W., Ferrington D. A. (2010). Mitochondrial DNA damage as a potential mechanism for age-related macular degeneration. *Investigative Ophthalmology & Visual Science*.

[21] Olsen T. W., Feng X. (2004). The Minnesota Grading System of eye bank eyes for age-related macular degeneration. *Investigative Ophthalmology & Visual Science*.

[22] Golestaneh N., Chu Y., Xiao Y. Y., Stoleru G. L., Theos A. C. (2017). Dysfunctional autophagy in RPE, a contributing factor in age-related macular degeneration. *Cell Death & Disease*.

[23] Dósa E., Heltai K., Radovits T. (2017). Dose escalation study of intravenous and intra-arterial N-acetylcysteine for the prevention of oto- and nephrotoxicity of cisplatin with a contrast-induced nephropathy model in patients with renal insufficiency. *Fluids and Barriers of the CNS*.

[24] Li Q., Dinculescu A., Shan Z. (2008). Downregulation of p22phox in retinal pigment epithelial cells inhibits choroidal neovascularization in mice. *Molecular Therapy*.

[25] Foresti R., Bucolo C., Platania C. M. B., Drago F., Dubois-Randé J. L., Motterlini R. (2015). Nrf2 activators modulate oxidative stress responses and bioenergetic profiles of human retinal epithelial cells cultured in normal or high glucose conditions. *Pharmacological Research*.

[26] Handa J. T. (2012). How does the macula protect itself from oxidative stress?. *Molecular Aspects of Medicine*.

[27] Kim M. H., Chung J., Yang J. W., Chung S. M., Kwag N. H., Yoo J. S. (2003). Hydrogen peroxide-induced cell death in a human retinal pigment epithelial cell line, ARPE-19. *Korean Journal of Ophthalmology*.

[28] Lu L., Hackett S. F., Mincey A., Lai H., Campochiaro P. A. (2006). Effects of different types of oxidative stress in RPE cells. *Journal of Cellular Physiology*.

[29] Chang C. C., Huang T. Y., Chen H. Y. (2018). Protective effect of melatonin against oxidative stress-induced apoptosis and enhanced autophagy in human retinal pigment epithelium cells. *Oxidative Medicine and Cellular Longevity*.

[30] Ray P. D., Huang B. W., Tsuji Y. (2012). Reactive oxygen species (ROS) homeostasis and redox regulation in cellular signaling. *Cellular Signalling*.

[31] Lin H., Xu H., Liang F. Q. (2011). Mitochondrial DNA damage and repair in RPE associated with aging and age-related macular degeneration. *Investigative Opthalmology & Visual Science*.

[32] O'Brien W. J., Heimann T., Rizvi F. (2009). NADPH oxidase expression and production of superoxide by human corneal stromal cells. *Molecular Vision*.

[33] Qiu Y., Tao L., Lei C. (2015). Downregulating p22phox ameliorates inflammatory response in Angiotensin II-induced oxidative stress by regulating MAPK and NF-*κ*B pathways in ARPE-19 cells. *Scientific Reports*.

[34] Yang J., Li J., Wang Q., Xing Y., Tan Z., Kang Q. (2018). Novel NADPH oxidase inhibitor VAS2870 suppresses TGF‑*β*‑dependent epithelial‑to‑mesenchymal transition in retinal pigment epithelial cells. *International Journal of Molecular Medicine*.

[35] Tu G., Zhang Y. F., Wei W. (2016). Allicin attenuates H2O2-induced cytotoxicity in retinal pigmented epithelial cells by regulating the levels of reactive oxygen species. *Molecular Medicine Reports*.

[36] Bedard K., Krause K. H. (2007). The NOX family of ROS-generating NADPH oxidases: physiology and pathophysiology. *Physiological Reviews*.

[37] Birben E., Sahiner U. M., Sackesen C., Erzurum S., Kalayci O. (2012). Oxidative stress and antioxidant defense. *World Allergy Organization Journal*.

[38] St-Pierre J., Drori S., Uldry M. (2006). Suppression of reactive oxygen species and neurodegeneration by the PGC-1 transcriptional coactivators. *Cell*.

[39] Iacovelli J., Rowe G. C., Khadka A. (2016). PGC-1*α* induces human RPE oxidative metabolism and antioxidant capacity. *Investigative Ophthalmology & Visual Science*.

[40] Kalinina E. V., Chernov N. N., Novichkova M. D. (2014). Role of glutathione, glutathione transferase, and glutaredoxin in regulation of redox-dependent processes. *Biochemistry*.

[41] Beatty S., Koh H. H., Phil M., Henson D., Boulton M. (2000). The role of oxidative stress in the pathogenesis of age-related macular degeneration. *Survey of Ophthalmology*.

[42] van Leeuwen R., Boekhoorn S., Vingerling J. R. (2005). Dietary intake of antioxidants and risk of age-related macular degeneration. *The Journal of the American Medical Association*.

[43] Li X. M., Huang D., Yu Q., Yang J., Yao J. (2018). Neuroligin-3 protects retinal cells from H_2_O_2_ -induced cell death via activation of Nrf2 signaling. *Biochemical and Biophysical Research Communications*.

[44] Du L., Chen J., Xing Y. Q. (2017). Eupatilin prevents H2O2-induced oxidative stress and apoptosis in human retinal pigment epithelial cells. *Biomedicine & Pharmacotherapy*.

[45] Li K. R., Yang S. Q., Gong Y. Q. (2016). 3H-1,2-dithiole-3-thione protects retinal pigment epithelium cells against ultra-violet radiation via activation of Akt-mTORC1-dependent Nrf2-HO-1 signaling. *Science Reports*.

[46] Wang K., Jiang Y., Wang W., Ma J., Chen M. (2015). Escin activates AKT-Nrf2 signaling to protect retinal pigment epithelium cells from oxidative stress. *Biochemical and Biophysical Research Communications*.

[47] Kagan D. B., Liu H., Hutnik C. M. (2012). Efficacy of various antioxidants in the protection of the retinal pigment epithelium from oxidative stress. *Clinical Ophthalmology*.

[48] Eichler W., Reiche A., Yafai Y., Lange J., Wiedemann P. (2008). Growth-related effects of oxidant-induced stress on cultured RPE and choroidal endothelial cells. *Experimental Eye Research*.

[49] Dilger R. N., Baker D. H. (2007). Oral N-acetyl-L-cysteine is a safe and effective precursor of cysteine. *Journal of Animal Science*.

[50] Kanter M. Z. (2006). Comparison of oral and i.v. acetylcysteine in the treatment of acetaminophen poisoning. *American Journal of Health-System Pharmacy*.

[51] Whyte A. J., Kehrl T., Brooks D. E., Katz K. D., Sokolowski D. (2010). Safety and effectiveness of acetadote for acetaminophen toxicity. *Journal of Emergency Medicine*.

[52] Clinicaltrials.gov/ct2/results?cond=&term=n-acetyl+cysteine

[53] Cotgreave I. A. (1997). N-acetylcysteine: pharmacological considerations and experimental and clinical applications. *Advances in Pharmacology*.

[54] Zafarullah M., Li W. Q., Sylvester J., Ahmad M. (2003). Molecular mechanisms of *N*-acetylcysteine actions. *Cellular and Molecular Life Sciences (CMLS)*.

[55] Laragione T., Bonetto V., Casoni F. (2003). Redox regulation of surface protein thiols: identification of integrin *α*-4 as a molecular target by using redox proteomics. *Proceedings of the National Academy of Sciences of the United States of America*.

[56] Franco R., Cidlowski J. A. (2012). Glutathione efflux and cell death. *Antioxidants & Redox Signaling*.

[57] Martínez de Toda I., Vida C., Garrido A., de la Fuente M. (2019). Redox parameters as markers of the rate of aging and predictors of life span. *The Journals of Gerontology: Series A*.

[58] Cohen S. M., Olin K. L., Feuer W. J., Hjelmeland L., Keen C. L., Morse L. S. (1994). Low glutathione reductase and peroxidase activity in age-related macular degeneration. *British Journal of Ophthalmology*.

[59] Qin L., Mroczkowska S. A., Ekart A., Patel S. R., Gibson J. M., Gherghel D. (2014). Patients with early age-related macular degeneration exhibit signs of macro- and micro-vascular disease and abnormal blood glutathione levels. *Graefe's Archive for Clinical and Experimental Ophthalmology*.

