# Supplementary material for: N-Acetyl-L-cysteine Protects Human Retinal Pigment Epithelial Cells from Oxidative Damage: Implications for Age-Related Macular Degeneration
Source: Oxid Med Cell Longev. 2019 Aug 14;2019:5174957. doi: 10.1155/2019/5174957 (PMC6710748; doi:10.1155/2019/5174957)
Supplement: Supplementary Materials — Figure S1: characterization of NAC. Figure S2: traces from Cell Mito Stress Test analysis of the oxygen consumption rate. Supplemental Table 1: donor gender and ages used in each figure. [file 5174957.f1.pdf]

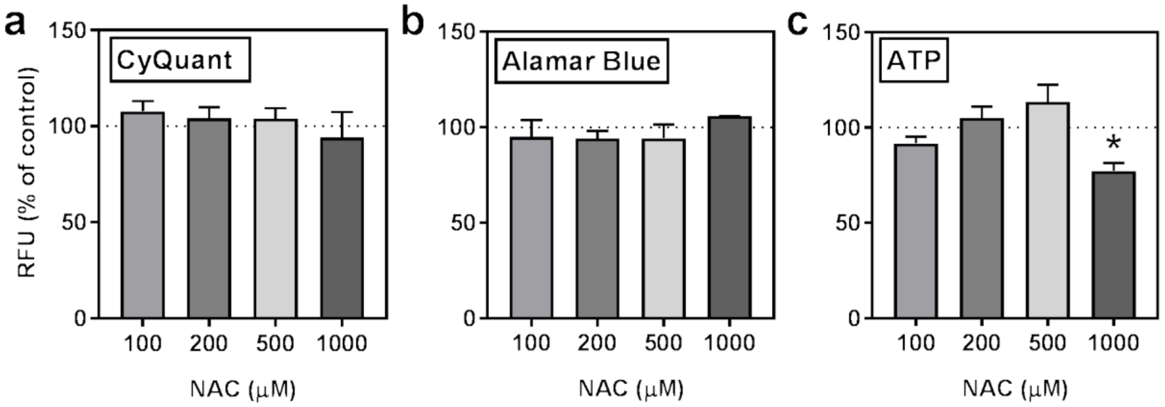

Supplemental Figure 1: Characterization of NAC treatment effect. RPE cells from donors without AMD (n = 3) were treated with NAC for 24hrs at a range of concentrations (100-1000μM). Cell viability (**a** and **b**) and ATP content (**c**) are expressed as percentage of no treatment control. Data are mean (± SEM). **p*<0.05 determined by One-Way ANOVA with Dunnett's post hoc.

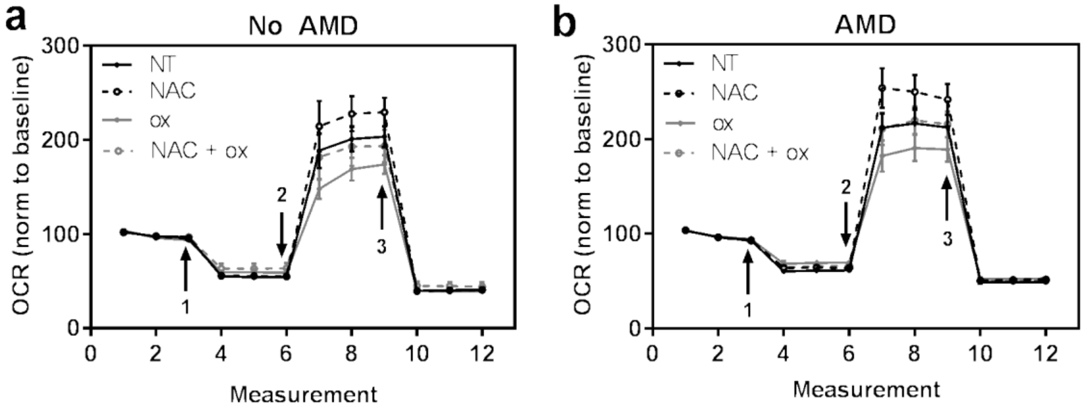

Supplemental Figure 2: NAC treatment improves mitochondrial function. Traces from Extracellular Flux Analyzer showing oxygen consumption rate (OCR) normalized to baseline for cells from No AMD (n=6) donors (**a**) and AMD (n=12) donors (**b)** with or without NAC pre-treatment and/or H_2O_2 (ox) treatment. NT = no treatment. Data are mean (± SEM). Arrows indicate injection of oligomycin (1), FCCP (2), and antimycin A and rotenone (3) to perturb mitochondrial function. Data from these traces were used to calculate the parameters of mitochondrial function found in Figure 6.

Supplement Table 1. Donor Characteristics and Clinical Information [A]

Figure	Disease State[B]	Sample (n)	Sex Male (n)	Sex Female (n)	Age [C] (Mean ± SD)
1a - 1e	No AMD	7	3	4	66 ± 10.1
	AMD	7	5	2	76 ± 6.8
1f & 1g	No AMD	6	5	1	60 ± 9.2
	AMD	8	6	2	73 ± 9.1
2	No AMD	5	2	3	69 ± 7.4
	AMD	10	6	4	75 ± 3.6
3	No AMD	6	2	4	65 ± 8.6
	AMD	15	10	5	72 ± 10.1
4	No AMD	6	2	4	65 ± 8.7
	AMD	12	9	3	73 ± 7.7
5	No AMD	6	3	3	67 ± 10.0
	AMD	7	5	2	69 ± 15.3
6	No AMD	6	4	2	59 ± 8.8
	AMD	11	7	4	70 ± 15.4

[A] Information supplied by Lions Gift of Sight (St. Paul, MN).

[B] Minnesota Grading System (MGS) was used to evaluate the stage of AMD in eye bank eyes (Olsen and Feng, 2004). No AMD = MGS1; AMD = MGS2, 3, or 4.

[C] There was no statistically significant difference in age between No AMD and AMD donors as determined by t-test comparison for donors used in each figure.
